# Processing‐Induced Metabolic Reprogramming of *Litsea coreana* Modulates Its Phytochemical Profile and Bioactivities

**DOI:** 10.1002/fsn3.71880

**Published:** 2026-05-13

**Authors:** Jie Li, Jinyan Zhao, Yaxin Liu, Xiuming Zhai, Fuliang Xiao, Dan Wang, Qiyang Chen

**Affiliations:** ^1^ Research Institute of Tea Chongqing Academy of Agricultural Sciences Chongqing China; ^2^ College of Life Sciences and Agri‐Forestry Southwest University of Science and Technology Mianyang China; ^3^ Tianfu Institute of Research and Innovation, Southwest University of Science and Technology Chengdu China

**Keywords:** α‐amylase inhibitory activity, antioxidant capacity, hawk tea, non‐targeted metabolomics

## Abstract

Hawk tea (*Litsea coreana)*, a traditional caffeine‐free herbal beverage, has gained increasing consumer interest owing to its health‐promoting properties. Yet, how processing shapes its metabolic composition and bioactivity is not fully elucidated. In this study, we investigated the chemical composition, metabolic profile, and in vitro bioactivity of three processed hawk teas: green (GHT), yellow (YHT), and black (BHT). Chemical analysis revealed a substantial reduction in phenolic and flavonoid contents during the processing of GHT into BHT, accompanied by a noticeable color evolution from yellow‐green to orange‐red. Non‐targeted metabolomic analysis uncovered 5163 metabolic features, with 2610 being significantly differential, primarily from flavonoids, phenolic acids, amino acids, and sugars. Molecular network analysis showed that phenylpropanoid biosynthesis and amino acid degradation were the central pathways remodeled during processing, driving the conversion of monomeric flavanols in GHT to complex polymeric pigments in BHT. This metabolic shift corresponded to a marked divergence in bioactivity, as GHT demonstrated the strongest antioxidant potential and exhibited the greatest α‐amylase inhibitory activity. By systematically delineating the metabolic landscape underlying hawk tea quality, our work provides a mechanistic understanding and a scientific basis for its tailored application in functional food products.

## Graphical abstract

This study employs untargeted metabolomics to investigate how processing methods (green, yellow, and black) reshape the phytochemical profile and bioactivities of hawk tea (*Litsea coreana*). The results reveal that the degree of fermentation drives a metabolic reprogramming from monomeric flavanols to complex polymeric pigments, with green hawk tea exhibiting the strongest antioxidant and α‐amylase inhibitory activities. Key differential metabolites, including flavonoids, phenolic acids, and anthocyanins, are significantly correlated with the divergence in bioactivity. These findings provide a metabolomic basis for the tailored processing of hawk tea as a functional beverage.

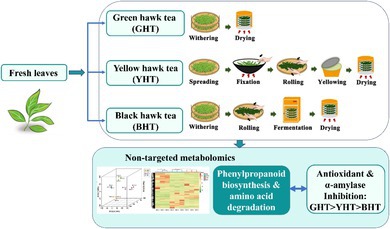

## 1 Introduction

Hawk tea (*Litsea coreana*) is a traditional decaffeinated herbal beverage widely consumed in southwestern China, such as Chongqing, Sichuan, and Yunnan, due to its unique sensory characteristics and potential health benefits. It is usually processed from the tender buds or shoots of *Litsea coreana*, mainly including two species: *L. coreana* var. lanuginosa and *L. coreana* var. sinensis (Zhao, Huang, et al. 2024). The production process includes high‐temperature enzyme fixation and kneading, which gives it a unique taste: fragrant and sweet, with a hint of camphor wood aroma, neither bitter nor astringent (Yu et al. 2016). These appealing attributes, together with its absence of caffeine, render it especially suitable for caffeine‐sensitive individuals, explaining its growing popularity and commercial success in recent years (Ma et al. 2011).

In addition to its taste and aroma, hawk tea has a long history of application in traditional Chinese medicine. The ancient book Compendium of Materia Medica records that it can help our body detoxify, eliminate edema, and quench thirst (Luo et al. 2023). Modern phytochemistry research has identified a series of bioactive components in hawk tea, including flavonoids, polysaccharides, and polyphenols (Feng et al. 2025), which are considered the material basis for its various pharmacological activities, such as antioxidant, anti‐inflammatory, and enzyme inhibition (Jiang et al. 2025). Of particular relevance to metabolic health, hawk tea polyphenols have shown promise in managing postprandial blood glucose by inhibiting key enzymes such as α‐glucosidase and α‐amylase (Lv et al. 2025). So far, many studies have been trying to figure out the function of a single component; For example, quercetin is considered to lower cholesterol (Feng et al. 2019), some flavonoids can protect the liver (Xu et al. 2022), and polysaccharide HTP‐1 has been found to regulate the immune system (Yu et al. 2023). Although these decomposition methods are very useful, they often overlook a key factor that changes the chemical composition of tea, namely the manufacturing process. This process triggers a series of biochemical transformations through enzymatic and non‐enzymatic reactions, fundamentally altering the structure and activity of bioactive compounds. Therefore, the systematic effects of processing methods on the sensory quality and plant chemical composition of hawk are still poorly understood.

Non‐targeted metabolomics has emerged as a powerful tool for deciphering food composition and metabolic dynamics. This comprehensive and unbiased approach is particularly suited for unraveling the complex metabolic profiles of plant‐based beverages, facilitating the understanding of taste evolution, tracking compositional shifts during processing, and aiding in final product quality assessment (Cheng et al. 2021). The utility of this methodology is exemplified in hawk tea research. For instance, one study integrated non‐targeted metabolomics with quantitative descriptive analysis (QDA) to differentiate hawk black tea from hawk white tea, revealing significant associations between nonvolatile metabolites and taste characteristics and identifying ten key flavonoids responsible for their taste differences (Zhao, Li, et al. 2024). Complementing this, another recent investigation further expanded our understanding of how intrinsic plant traits influence tea quality. By examining hawk black tea made from green, purple, and mixed leaves, it was revealed that leaf color significantly shapes the flavor profile and underlying metabolite composition, with purple‐leaf tea exhibiting prominent floral‐fruity notes and green‐leaf tea displaying fresh camphoraceous characteristics (He et al. 2025). This underscores the pivotal role of both raw material properties and processing in defining the final product. While the health benefits of hawk tea are recognized and the impact of leaf color on sensory quality is becoming clearer, a systematic investigation into the metabolic recombination and corresponding bioactivity shifts induced by different processing methods remains unexplored. Therefore, this study utilized *L. coreana* var. lanuginosa to produce three representative processing types: green, yellow, and black hawk tea. By integrating non‐targeted metabolomics with biochemical assays, we aim to delineate the metabolic reprogramming during processing, correlate these changes with antioxidant capacity and α‐amylase inhibitory activity, and reveal the underlying chemical mechanisms for the observed divergence in bioactivities. Our findings are expected to establish a metabolic basis for setting quality standards and promoting the high‐value utilization of hawk tea resources.

## 2 Materials and Methods

### 2.1 Sample Collection and Preparation

**FIGURE 1:**
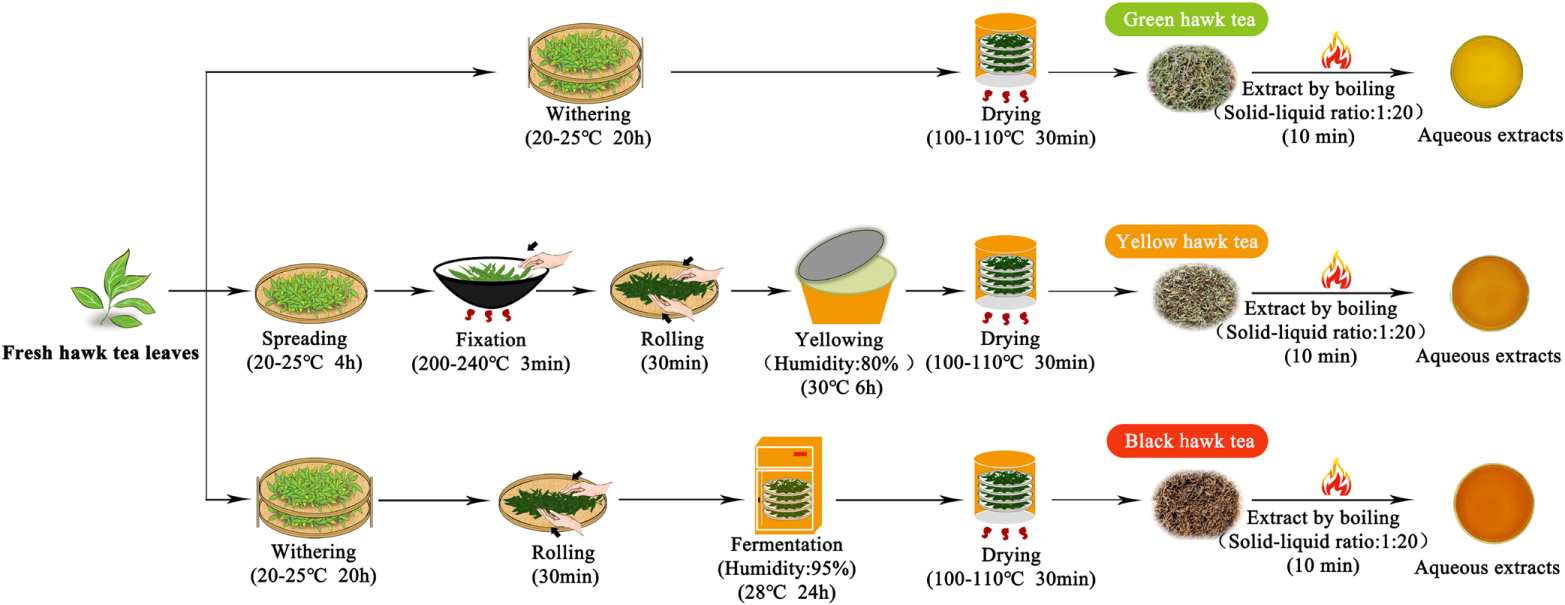
Schematic diagram of the manufacturing processes for green (GHT), yellow (YHT), and black hawk tea (BHT).

Fresh leaves of *L. coreana* var. lanuginosa with uniform maturity and quality grade were obtained in April 2024 from Youyang County, Chongqing. The specific tea processing conditions were determined based on standardized methodologies established in traditional hawk tea production practices and optimized through preliminary experiments to ensure representative product quality (Liu et al. 2020). According to the standard procedures (Figure 1), these leaves are processed into three different kinds of hawk tea: green (GHT), yellow (YHT), and black (BHT). The specific steps are as follows:

**GHT:** The fresh leaves were gently wilted at 20°C–25°C for 20 h, then baked directly at 100°C–110°C for 30 min to inactivate enzymes and obtain unfermented products.

**YHT:** The fresh leaves were spread at 20°C–25°C for 4 h, then fixed at 200°C–250°C for 3 min to inactivate enzymes. After cooling, the leaves were kneaded for 30 min, and then the leaves underwent a critical “yellowing” step, which is to be stacked at 30°C and 80% relative humidity for 6 h to promote slight fermentation. Finally, YHT was obtained by drying at 100°C–110°C for 30 min.

**BHT:** To produce fully fermented BHT, the leaves were first naturally withered at 20°C–25°C for 20 h. Subsequently, they were rolled for 30 min and then fermented at 28°C and 95% relative humidity for 24 h. The final step was drying at 100°C–110°C for 30 min.

### 2.2 Analysis of Primary Chemical Components

The contents of moisture, dry matter, and water extractable were determined according to the method of Zhao, Huang, et al. (2024). The soluble sugar content, free amino acid content, and total protein content were quantified using the sulfuric acid‐anthrone method (Yemm and Willis 1954), ninhydrin colorimetric method (Lee and Takahashi 1966), and Bradford assay (Bradford 1976), respectively. Tea pigments (theaflavins, thearubigins, and theabrownins) were determined according to our previously reported method (Li, Chen, et al. 2025).

### 2.3 Determination of Total Phenols and Total Flavonoids

The extraction of total phenolic content (TPC) and total flavonoid content (TFC) were performed according to the method of Zhao, Li, et al. (2024) with minor modifications. Briefly, 80 mg of each powdered tea sample was homogenized with 4 mL of 70% (v/v) aqueous methanol for 1 min, followed by ultrasonic‐assisted extraction for 50 min. The resulting mixture was then centrifuged at 3500*g* for 10 min. The supernatant was collected for determination of total phenols, total flavonoids, and antioxidant activity.

TPC was determined using the Folin–Ciocalteu colorimetric method (Wang, Li, et al. 2025). An aliquot of 100 μL of the extract was mixed with 900 μL of distilled water and 5 mL of 0.1 N Folin–Ciocalteu reagent. After standing for 5 min, 4 mL of a 75 g/L sodium carbonate (Na_2_CO_3_) solution was added. The reaction mixture was incubated in the dark at room temperature for 1 h, and the absorbance was measured at 765 nm using a T6‐1650F UV–Vis spectrophotometer (Puxi General Instrument Co. Ltd., Beijing, China). TPC was calculated based on a calibration curve prepared with gallic acid standards and expressed as milligrams of gallic acid equivalents per gram of dry weight (mg GAE/g DW).

TFC was measured using aluminum chloride (AlCl_3_) colorimetric method (Wang, Peng, et al. 2025). Briefly, 0.4 mL of extract, 1.6 mL of distilled water, and 0.4 mL of 5% (w/v) sodium nitrite (NaNO_2_) solution were mixed and reacted for 6 min. Subsequently, 0.4 mL of 10% (w/v) aluminum chloride solution was added and incubated for another 5 min. Finally, 1.6 mL of 4% (w/v) sodium hydroxide (NaOH) solution and 5.6 mL of distilled water were added in the mixture. The absorbance was determined at 510 nm. TFC is quantified using the rutin standard curve and expressed as milligrams of rutin equivalent per gram of dry weight (mg RE/g DW).

### 2.4 Metabolite Extraction and LC–MS/MS Analysis

The extraction of metabolites is done according to the method provided by Biomarker Technologies Corporation (Li, Wen, et al. 2025). Briefly, 50 mg of each powdered sample was accurately weighed and extracted with 1 mL of a pre‐cooled methanol/acetonitrile/water mixture (2:2:1, v/v/v) containing 2‐chloro‐l‐phenylalanine as an internal standard (20 mg/L). The mixture was homogenized with grinding beads at 45 Hz for 10 min, followed by sonication in an ice‐water bath for 10 min. After incubation at −20°C for 1 h, the samples were centrifuged at 12,000 rpm for 15 min at 4°C. The supernatant was collected, and the extraction procedure was performed once. The combined supernatant was transferred and dried under vacuum. The dried metabolites were reconstituted in 160 μL of acetonitrile/water (1:1, v/v), vortexed for 30 s, sonicated in an ice‐water bath for 10 min, and centrifuged again under the same conditions. Finally, 120 μL of the supernatant was transferred to a vial for UHPLC–MS/MS analysis. Metabolomic analysis was performed using three independent biological replicates per group (*n* = 3), with all samples randomized during the extraction and instrumental analysis sequence to control for batch effects. A quality control (QC) sample was prepared by pooling 10 μL of supernatant from each sample.

### 2.5 LC–MS/MS Data Acquisition

Chromatographic separation was achieved using a Waters Acquity UPLC HSS T3 column (100 mm × 2.1 mm, 1.8 μm) maintained at ambient temperature. The mobile phase consisted of (A) water with 0.1% formic acid and (B) acetonitrile with 0.1% formic acid. The gradient elution program was set as follows: 0–0.5 min, 5% B; 0.5–5.5 min, 5%–50% B; 5.5–9.0 min, 50%–95% B; 9.0–10.5 min, 95% B; 10.5–12.0 min, 95%–5% B; 12.0–14.0 min, 5% B, at a constant flow rate of 0.4 mL/min. The injection volume was 2 μL.

Mass spectrometry detection was performed on a Waters Xevo G2‐XS QToF spectrometer operating in MSe mode under the control of MassLynx V4.2 software. The acquisition method cycled between low collision energy (0 eV) and a ramped high collision energy range (10–40 eV), with a scan time of 0.2 s per spectrum. The ESI source parameters were set as follows: capillary voltage: 2500 V (positive) or −2000 V (negative); cone voltage: 30 V; source temperature: 100°C; desolvation temperature: 500°C; cone gas flow: 50 L/h; and desolvation gas flow: 800 L/h. The mass scan range was m/z 50–1200.

### 2.6 Data Preprocessing and Metabolite Annotation

The raw LC–MS data were processed using Progenesis QI software (Waters) for peak picking, alignment, and normalization. Metabolite annotation was performed according to the confidence levels defined by the Metabolomics Standards Initiative (MSI). Putative annotation (Level 2–3) was initially performed by querying the accurate mass (mass tolerance < 20 ppm) against the METLIN database and a custom in‐house library. For metabolites where MS/MS spectra were available, Level 2 annotation (probable structure) was assigned based on spectral matching. All annotations are considered putative unless otherwise validated with authentic standards.

### 2.7 Multivariate Statistical and Pathway Analysis

The normalized peak area data matrix (normalized to total ion current in Progenesis QI, log‐transformed, and Pareto‐scaled) was imported into the R environment for multivariate statistical analysis. Missing values were imputed with half of the minimum positive value for each metabolite. Principal component analysis (PCA) was performed to visualize natural clustering and assess QC sample reproducibility. To identify differentially abundant metabolites, an orthogonal partial least squares‐discriminant analysis (OPLS‐DA) model was constructed using the ropls R package. The model was rigorously validated through 7‐fold cross‐validation, and its robustness was assessed via permutation testing (*n* = 200) to obtain R^2^Y, Q^2^ values and the corresponding permutation test intercepts (Figure S1). Metabolites with a variable importance in projection (VIP) score > 1.0, a fold change (FC) > 2.0 or < 0.5, and a Student's *t*‐test *p*‐value < 0.05 with a false discovery rate (FDR) *q*‐value < 0.2 were considered significantly differential. Metabolic pathway analysis was carried out using the Kyoto Encyclopedia of Genes and Genomes (KEGG, http://www.kegg.jp) and MetabolAnalyst4.0 (http://www.metaboanalyst.ca/).

### 2.8 Antioxidant Activities Assays

The antioxidant activities were determined using hot‐water infusions of the tea samples to simulate common consumption. The antioxidant activities (ABTS, DPPH, and FRAP) were determined, referring to previously reported by Wang, Peng, et al. (2025). In the ABTS experiment, a 7 mM ABTS stock solution with 140 mM potassium persulfate was mixed overnight to generate a free radical cation, and then diluted to an absorbance of 0.70 ± 0.02 at 734 nm. Hawk tea extract (40 μL) was reacted with 3 mL of diluted ABTS solution at 30°C for 10 min, and the absorbance was obtained at the wavelength of 734 nm. For the DPPH assay, 100 μL of the extract and 3.5 mL of DPPH solution (75 μmol/L) were mixed and incubated in the dark at room temperature for 30 min. The absorbance was recorded at 517 nm. For the FRAP assay, the working reagent was prepared freshly by mixing 300 mM acetate buffer (pH 3.6), 10 mM TPTZ solution in 40 mM HCl, and 20 mM FeCl_3_·6H_2_O solution in a 10:1:1 (v/v/v) ratio. Then, 200 μL of the extract was mixed with 3.8 mL of the FRAP reagent and incubated at 20°C for 30 min. The absorbance was measured at 593 nm. The antioxidant capacity was calculated based on the corresponding Trolox standard curve and expressed as micromoles of Trolox equivalents per gram of dry weight (μmol TE/g DW).

### 2.9 α‐Amylase Inhibitory Activity Assay

The inhibitory activities against α‐amylase were assessed according to the method of Chen et al. (2025) with slight adjustments. Briefly, hot‐water extracts were first prepared, then freeze‐dried to obtain a solid powder. This powder was then re‐dissolved and serially diluted to six concentrations (0.25, 0.5, 1, 2, 3, and 4 mg/mL). For α‐amylase inhibition, 120 μL of extract (or buffer as the negative control, or acarbose solution as the positive control) was mixed with 280 μL of 0.1 M phosphate buffer (pH 6.9) containing porcine pancreatic α‐amylase solution (50 U/mg; Macklin, China) at a final concentration of 0.5 U/mL and incubated at 37°C for 10 min. Then, 400 μL of 0.5% starch solution was added, and the reaction was stopped after another 10 min by adding 400 μL of DNS reagent. The mixture was boiled, cooled, diluted, and the absorbance was measured at 540 nm. The inhibitory percentage was calculated based on the Equation (1). The IC_50_ value (concentration causing 50% inhibition) was determined by fitting the dose–response data to a four‐parameter logistic model (or non‐linear regression) using GraphPad Prism software.

(1) $$\alpha -\text{amylase inhibitory activity}\;\left(\%\right)=\frac{A_{\text{Control}}-A_{\text{Sample}}}{A_{\text{Control}}}\times 100\%$$

### 2.10 Statistical Analysis

All chemical and bioactivity assays were conducted in at least three independent replicates. Data are presented as mean ± standard deviation (SD). Differences among the three tea types were analyzed by one‐way ANOVA followed by Tukey's post hoc test using IBM SPSS Statistics 25. A *p*‐value < 0.05 was considered statistically significant. The multivariate statistical methods applied to the metabolomics data are described in Section 2.7.

## 3 Results and Discussion

### 3.1 Chemical Composition

**FIGURE 2:**
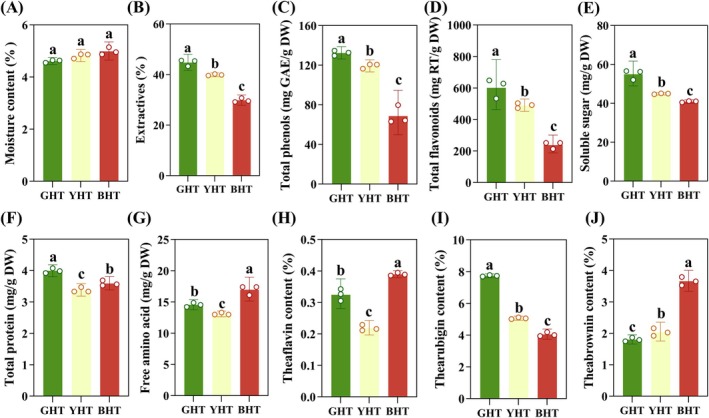
Primary chemical composition of green (GHT), yellow (YHT), and black hawk tea (BHT). Determined parameters include: (A) moisture content, (B) water extractables, (C) total phenolic content, (D) total flavonoid content, (E) soluble sugar, (F) total protein, (G) free amino acids, (H) theaflavin, (I) thearubigin, (J) theabrownin. Different lowercase letters indicate statistically significant differences among tea types (*p* < 0.05).

The processing method could strongly alter the phytochemical composition (primary and secondary metabolites) of hawk tea, thus affecting the sensory quality and health‐promoting functions (Jiang et al. 2025). The moisture content of the three processed hawk teas (GHT, YHT, and BHT) was determined, and all samples exhibited low levels below 5% (Figure 2A), indicating adequate drying during processing. There was a significant progressive decline in the contents of extracts, soluble sugars, total phenols, and total flavonoids as processing intensity increased (from GHT to YHT to BHT; Figure 2B–E). Notably, the total protein content from high to low was GHT, BHT, and YHT (Figure 2F). These findings suggest that the minimal processing in GHT best preserves the original chemical composition of hawk tea, whereas the progressive fermentation in YHT and BHT production drives significant metabolic conversion and potential loss (Wang et al. 2018). It is worth noting that the soluble sugars in YHT and BHT decreased by 18% and 26% respectively compared to GHT (Figure 2E), which can be attributed to their role as precursors in the Maillard reaction and their consumption during fermentation, which is crucial for the development of characteristic aromas (Wang, Shen, et al. 2022; Wang, Yu, et al. 2022).

Similar to soluble sugars, the conversion of phenolic compounds is particularly significant and directly related to the color of tea soup (Feng et al. 2025). The tea soups of GHT, YHT, and BHT are bright pale‐yellow green, bright orange, and deep orange red, respectively (Figure 1). This color change is a direct manifestation caused by processing‐induced metabolic remodeling. As previously reported, fermentation promotes the oxidation and polymerization of catechins (such as epicatechin, epigallocatechin, and epigallocatechin gallate) catalyzed by polyphenol oxidase and peroxidase, leading to a decrease in their content and the simultaneous formation of colored pigments such as theaflavins and thearubigins (Liu et al. 2020). In our study, the highest theaflavin content (0.38%) was found in completely fermented BHT, which is consistent with the expectation that a full fermentation process maximizes the enzymatic oxidation of catechins, thus following the known biochemical pathway for theaflavin formation established in black tea processing (Tanaka et al. 2001; Wang et al. 2017). Studies have shown that the detection of theaflavins (0.35%) in GHT may be a result of post‐harvest stress oxidation (Liao et al. 2019), while the lower‐than‐expected content of theaflavins in YHT suggests that its unique “yellowing” process may be beneficial for alternative metabolic pathways.

### 3.2 Characterization of Nonvolatile Metabolites in the Samples

#### 3.2.1 Global Analysis

**FIGURE 3:**
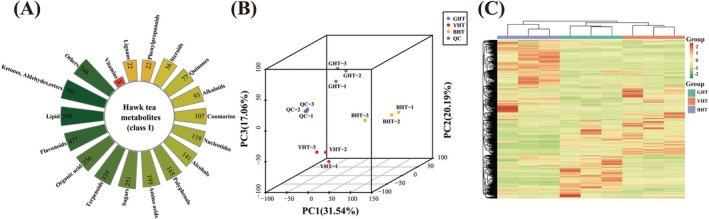
Non‐targeted metabolomic profiling of green (GHT), yellow (YHT), and black hawk tea (BHT). (A) Distribution of metabolite classes identified in hawk tea; unclassified or minor metabolites are grouped as “Others”. (B) Principal component analysis (PCA) score plot showing distinct metabolic clustering of GHT, YHT, and BHT. (C) Hierarchical clustering heatmap of metabolites across the three tea types.

We first compared the metabolite profiles of GHT, YHT, and BHT samples to reveal the processing‐induced metabolic reprogramming using a widely targeted metabolomic analysis. As shown in Figure 3A, a total of 5163 metabolic features were detected and putatively annotated in the positive and negative ion modes, categorized into 18 major classes. Among these, flavonoids, phenolic acids, amino acids, and sugars are the predominant compounds, indicating that they are crucial in shaping the characteristics of hawk tea (Yang et al. 2024). In addition, a large amount of ketones, aldehydes, and esters can serve as precursors for volatile substances, which may be released during the withering and drying stages of the processing by activating endogenous hydrolytic enzymes (Feng et al. 2025; Han et al. 2025).

Multivariate statistical analysis clearly demonstrated the global metabolic differences caused by processing. Principal component analysis (PCA) score plots revealed the similarities and differences among GHT, YHT, and BHT samples (Figure 3B), and quality control (QC) samples were closely clustered, indicating that the results of the metabolic profiles were reproducible and reliable. The first three main components (PC1 = 31.54%, PC2 = 20.19%, PC3 = 17.06%) explained 68.79% of the total variability, effectively capturing metabolic variance and clearly separating BHT from GHT and YHT along PC1. The obvious separation of BHT suggested that the fermentation process substantially altered the metabolite composition of hawk tea. Hierarchical cluster analysis (HCA) further confirmed metabolic differences, with GHT and YHT forming a major cluster, while BHT remained isolated (Figure 3C), strengthening the findings of PCA.

#### 3.2.2 Differences in Nonvolatile Metabolites

**FIGURE 4:**
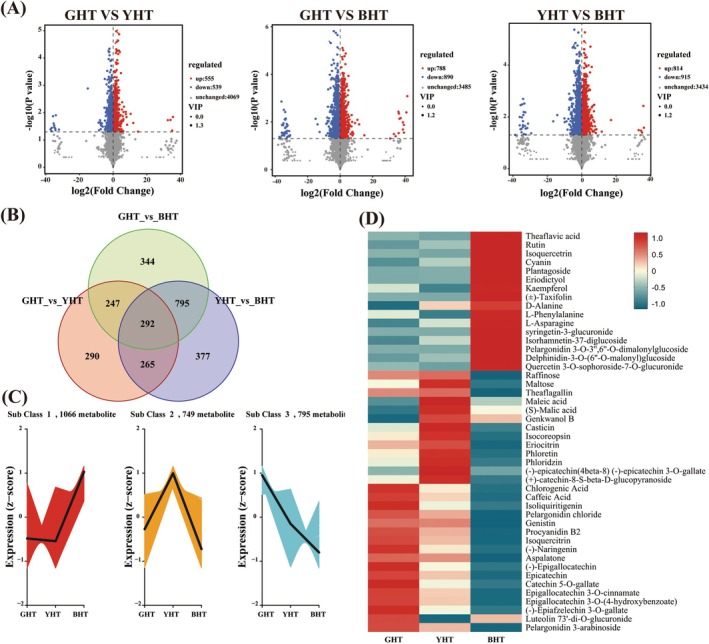
Differential metabolite analysis among green (GHT), yellow (YHT), and black hawk tea (BHT). (A) Volcano plots of differentially expressed metabolites (DEMs) from pairwise comparisons. (B) Venn diagram illustrating the overlap of DEMs across comparisons. (C) K‐means clustering of DEMs showing three distinct accumulation patterns. (D) Representative DEMs from each cluster.

In order to rapidly and accurately analyze differences in the metabolite compositions between pairwise comparison combination samples (three groups) to effectively screen for differential metabolites (DAMs), OPLS‐DA was employed (Figure S1). All OPLS‐DA models demonstrated excellent performance with clear group separation (as shown in the score plots of Figure S1), high goodness‐of‐fit (*R* ^2^ *Y*), and robust predictive ability (*Q* ^2^). Pairwise comparisons resulted in 1094 (GHT vs. YHT), 1678 (GHT vs. BHT), and 1729 (YHT vs. BHT) DAMs, respectively (Figure 4A). Overall, a total of 2610 unique putatively annotated metabolites underwent changes, and 292 core metabolites were shared among all comparisons (Table S1 and Figure 4B), including 48 types of flavonoids, 27 types of organic acids, 11 types of terpenoids, 12 types of amino acids, and other secondary metabolites such as polyphenols and lipids. These shared metabolites form the basis of a universal metabolic network activated by processing stress. Through pairwise comparative analysis, 290, 377, and 344 unique DAMs were identified in GHT, YHT, and BHT, respectively (Figure 4B). These unique DAMs may constitute the specific chemical properties and material basis of the sensory and functional characteristics unique to three types of hawk tea.

The annotated secondary differential metabolites (2610 DAMs) were divided into three different clusters through K‐means (Figure 4C,D) classification to determine the metabolite content changes across the samples. Subclass 1, which contained 1066 secondary differential metabolites, was highly enriched in BHT. These metabolites included flavonoids such as rutin, isoquercetrin, eriodictyol, kaempferol, and (±)‐taxifolin, which deepen the color of hawk tea and contribute to its mellow taste and health efficacy in anti‐cholesterol (Ma et al. 2025). Moreover, the content of amino acids such as l‐valine, l‐phenylalanine, d‐alanine, and l‐asparagine was significantly higher in the BHT samples, which is an outcome that was consistent with the free amino acid content (Figure 2G). The sweetness of fermented hawk tea is derived from soluble sugars and sweet amino acids, including d‐alanine and l‐asparagine (Feng et al. 2025; Yu and Yang 2019). Subclass 2 contained 749 metabolites, such as eriocitrin, phloretin, (S)‐malic acid, maleic acid, theaflagallin, maltose, raffinose, and steviolmonoside, which were specifically enriched in YHT. Maltose, raffinose, and steviolmonoside provided the sweetness, while (S)‐malic acid brings a refreshing sourness, resulting in a vibrant and well‐balanced tea soup (Shevchuk et al. 2020). Theaflagallin and some flavonoids (eriocitrin and phloretin) are the major contributors that affect astringency and bitter taste of tea, but they also endow it with healthy properties, such as antioxidant, anti‐inflammatory, and cardiovascular protection (Zhao, Huang, et al. 2024). Subclass 3 comprised 795 secondary metabolites, such as chlorogenic acid, caffeic acid, (−)‐epigallocatechin, procyanidin b2, isoquercitrin, and epicatechin, which are preserved due to the inactivation of oxidase. The processing of GHT was markedly simpler than that of YHT and BHT, centered on a “fixation” step to inactivate most polyphenol oxidases and excluding prolonged high‐temperature treatment and fermentation; this resulted in a tea with a comparatively strong, fresh, and refreshing profile (Liu et al. 2020). The significant enrichment of these specific phenolic acids and flavan‐3‐ols underscores the success of the fixation process in preserving the characteristic fresh‐tasting compounds in GHT. In summary, the clustering of metabolites effectively delineates the distinct biochemical basis for the unique sensory and functional properties of the three hawk tea types.

#### 3.2.3 Regulatory Network Analysis

**FIGURE 5:**
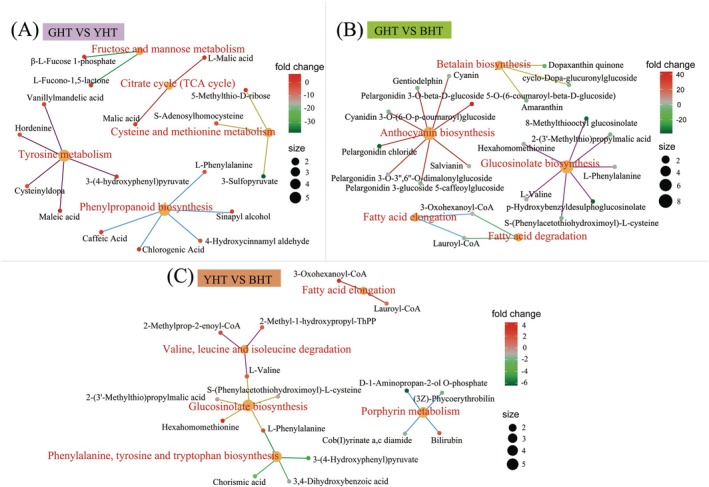
Regulatory network analysis of differentially expressed metabolites (DEMs) in green (GHT), yellow (YHT), and black hawk tea (BHT). Significantly enriched pathways are displayed, highlighting the metabolic shifts driven by processing.

To elucidate the chemical diversity of metabolites and their accumulation patterns in hawk black tea derived from different processing methods, a network enrichment analysis based on DAMs to reflect the intersection between metabolic pathways and potential target enzymes or metabolites under different processing methods (Figure 5 and Table S2). Five key metabolic pathways were identified for each group through inter group comparisons of GHT and YHT, GHT and BHT, and YHT and BHT. These pathways constitute a regulatory network that is associated with distinct transformation of hawk tea from its initial form (GHT) into two unique products derived from different processing routes: YHT and BHT. As shown in Figure 5A, “fructose and mannose metabolism”, “TCA cycle”, “phenylpropanoid biosynthesis”, “cysteine and methionine metabolism”, and “tyrosine metabolism” were the principal metabolic processes in the comparison between GHT and YHT. These processes notably enhanced the production of metabolites such as malic acid, chlorogenic acid, caffeic acid, maleic acid, and l‐phenylalanine. Among these components, the accumulation of TCA cycle intermediates (such as malic acid) provides a direct sour and refreshing taste to the tea infusion (Shevchuk et al. 2020). Meanwhile, the synergistic interaction between the phenylpropanoid pathway (chlorogenic acid and caffeic acid) and amino acid metabolism (l‐phenylalanine) not only contributes to the formation of refreshing flavor precursors, but also suggests that the mild fermentation process used in YHT may trigger the transformation and accumulation of flavor compounds through moderate microbial or enzymatic activity, while successfully preserving the fresh character resulting from the enzyme inactivation step (Sun et al. 2024). Interestingly, “anthocyanin biosynthesis”, “betalain biosynthesis”, “glucosinolate biosynthesis”, “fatty acid elongation and degradation” were the dominant processes in the correlation network comparison between GHT and BHT. These pathways contributed significantly to the development of BHT's characteristic dark color and complex flavor profile, as evidenced by the accumulation of key pigments such as pelargonidin 3‐O‐3″,6″‐O‐dimalonylglucoside, pelargonidin 3‐glucoside 5‐caffeoylglucoside, and cyanidin 3‐O‐(6‐O‐*p*‐coumaroyl)glucoside. This metabolic profile suggests that the distinctive reddish‐brown hue of BHT may result from anthocyanin transformation, while its characteristic baked and sweet aroma could arise from the degradation of fatty acids and sulfur‐containing precursors, contributing to a flavor profile richer than that of GHT (Liu et al. 2025; Zhao, Li, et al. 2024). The comparison between YHT and BHT underscored significant differences in the pathways of “valine, leucine and isoleucine degradation”, “fatty acid elongation”, “glucosinolate biosynthesis”, “phenylalanine, tyrosine and tryptophan biosynthesis”, and “porphyrin metabolism” with metabolites such as l‐valine and lauroyl‐CoA indicating that branched chain amino acid breakdown metabolism and lipid metabolism are particularly active during the transition from mild fermentation to complete fermentation, which may contribute to the production of aromatic compounds.

### 3.3 Global Visualization of Flavonoid Biosynthesis Pathway Changes

**FIGURE 6:**
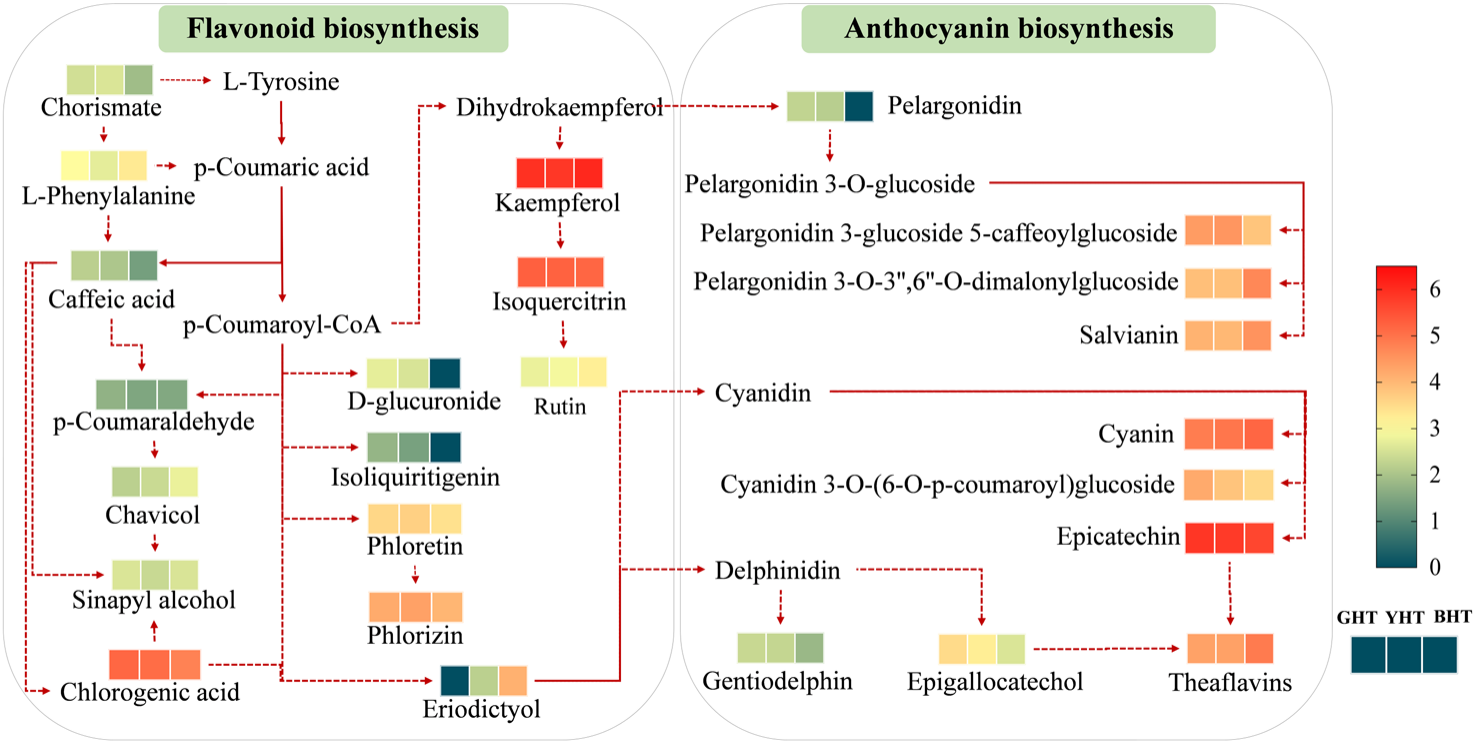
Comparative visualization of metabolite levels in phenylpropanoid biosynthesis pathway. Metabolites labeled in red or bold blue indicate significant up‐ or down‐regulation, respectively, in the specified comparisons.

In order to systematically elucidate the remodeling effects of different processing methods on the metabolism of flavonoids in hawk tea, we constructed a comprehensive metabolic regulatory network based on DAMs and pathway enrichment analysis (Figure 6, Table S3). The network reveals that the processing of GHT, YHT, and BHT shares a metabolic reprogramming framework centered on the flavonoid and anthocyanin biosynthesis pathways, distinguishing the three types of hawk tea (Feng et al. 2025). The results outline a clear metabolic trajectory from simple flavonoid monomers to complex polymeric pigments (Zhao et al. 2019). In GHT, rapid enzyme inactivation effectively preserves monomeric flavan‐3‐ols, such as catechins and epigallocatechin gallate, laying the foundation for its fresh taste and health potential (Feng et al. 2025); in contrast, BHT undergoes extensive enzymatic and non‐enzymatic polymerization during full fermentation (Liu et al. 2025), resulting in the substantial accumulation of complex pigments such as theaflavins, thearubigins, and anthocyanins (e.g., pelargonidin‐3‐glucoside). This transformation is not only a quantitative accumulation but also a qualitative leap, significantly enriching the flavor layers and biological activity diversity of the tea soup. YHT, which is in an intermediate metabolic state, has its unique ‘steamed yellow’ process precisely regulating the metabolic flux of the flavonoid pathway. It both inhibits deep pigment conversion similar to BHT and initiates limited and specific precursor transformations, thereby developing a golden‐yellow soup color and a unique mellow and refreshing taste while preserving the fresh flavor compounds of GHT (Sun et al. 2024).

Further pathway enrichment analysis highlighted key regulatory nodes in the flavonoid biosynthesis network. Comparisons between GHT and BHT showed that the anthocyanin biosynthesis pathway was significantly activated, with metabolites such as pelargonidin 3‐O‐3″,6″‐O‐dimalonylglucoside markedly upregulated, whereas comparisons between GHT and YHT indicated enhanced phenylpropanoid biosynthesis activity, with intermediates like chlorogenic acid and caffeic acid providing crucial precursors for the formation of the flavonoid backbone. These findings directly link specific processing‐induced metabolic changes to the distinctive color, taste, and functional properties of different types of hawk tea. Overall, this regulatory network resembles a metabolic map, illustrating that from the rapid de‐enzyming of GHT and the controlled yellowing of YHT to the full fermentation of BHT, each processing step acts as a metabolic switch, directing biochemical precursors—especially within the flavonoid biosynthesis pathway—into different metabolic trajectories, ultimately producing three hawk tea products with distinct chemical compositions, sensory characteristics, and functional attributes.

### 3.4 Antioxidant Activity and α‐Amylase Inhibition

**FIGURE 7:**
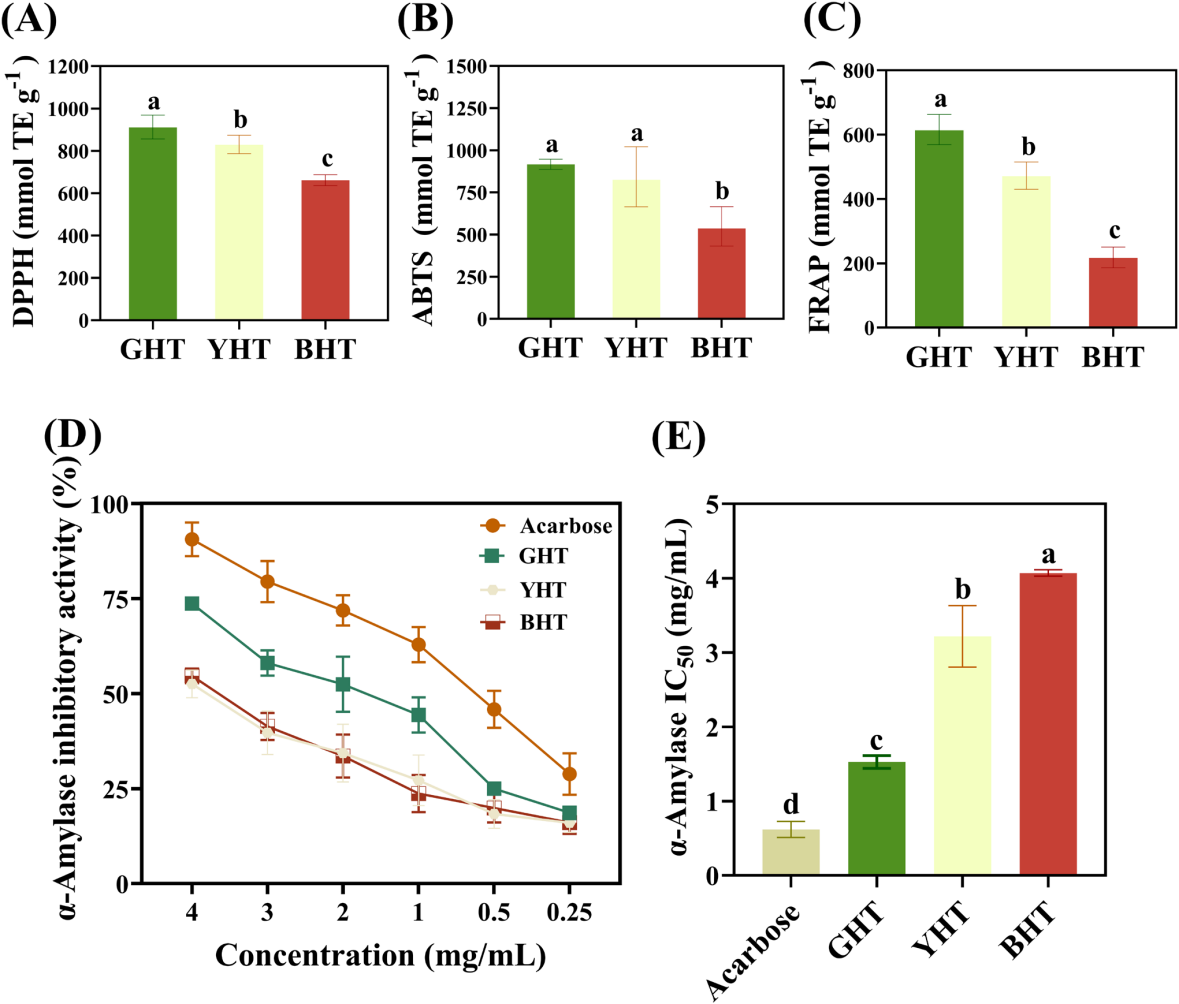
In vitro bioactivity assessment of green (GHT), yellow (YHT), and black hawk tea (BHT). Antioxidant capacity evaluated by (A) ABTS, (B) DPPH, and (C) FRAP assays; (D) α‐amylase inhibitory activity. Different letters denote significant differences (*p* < 0.05).

The in vitro antioxidant activities (DPPH, ABTS, and FRAP) of GHT, YHT, and BHT were measured. As shown in Figure 7A–C, the GHT sample exhibited the strongest DPPH and ABTS radical scavenging ability, as well as the superior iron reduction ability, which was consistent with Liu et al. (2020), who found that the minimal processing involved in GHT production optimally preserves a wide spectrum of native antioxidants (such as epigallocatechin, epigallocatechin gallate, and chlorogenic acid). Moreover, the exceptional antioxidant capacity of GHT is largely due to its high concentration of monomeric and glycosylated flavonoids, such as isoquercitrin and kaempferol, which are known to effectively neutralize free radicals (Chang et al. 2025). The overall antioxidant effect may also be amplified by synergistic interactions among the diverse phenolic compounds present, a phenomenon supported by existing literature (Jiang et al. 2025). On the contrary, the full fermentation process used for BHT promotes extensive oxidation and polymerization of these native phenolics. Although these transformations are essential to form the distinct color and flavor that characterize BHT, they also reduce its capacity to scavenge free radicals directly (Sun et al. 2024).

Consistent with its antioxidant potency, GHT also demonstrated the most potent inhibitory effect on α‐amylase (IC_50_ = 1.54 mg/mL), significantly outperforming both YHT and BHT (Figure 7D,E). This strong inhibitory activity aligns with findings that green tea extracts, rich in catechins like epicatechin gallate and epigallocatechin gallate, act as effective non‐competitive inhibitors of human pancreatic α‐amylase (Miao et al. 2015). The mechanism is structure‐dependent: specific polyphenols, including quercetin, proanthocyanidins, and tannins, influence starch digestibility by promoting resistant starch formation. Phenolic acids such as gallic acid disrupt starch structure via noncovalent interactions, with its high hydroxyl group content contributing to strong inhibition (Chen et al. 2025). Chlorogenic acid and its derivatives enhance inhibition through lipophilicity (Wang, Li, et al. 2022) and function as mixed‐type inhibitors by forming hydrogen bonds with α‐amylase, thereby altering its secondary structure (Zheng et al. 2020). Other phenolics like apigenin‐7‐glucoside also show significant α‐amylase inhibitory capacity (Witkowska‐Banaszczak et al. 2020), while acids including coumaric and caffeic acid suppress enzyme activity by interfering with carbohydrate metabolic pathways.

### 3.5 Relationship Between Metabolites and Biological Properties

**FIGURE 8:**
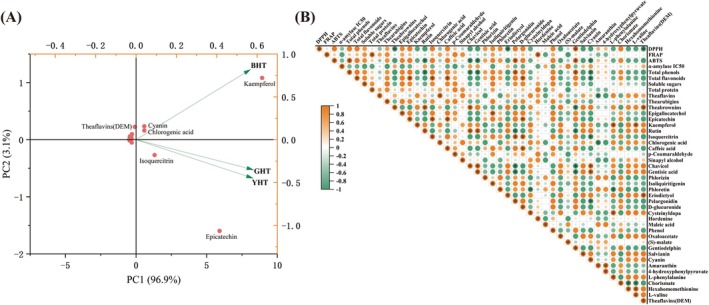
Correlation analysis between metabolite profiles and bioactivities. (A) Biplot from double‐standardized matrix of hawk tea metabolites. (B) Correlation heatmap between key chemical components and biological activities (antioxidant and α‐amylase inhibition).

Distinct processing methods modulate biological activity by differentially reshaping the chemical profile of the final product (Figure 8). Correlation analysis revealed that total phenolic content and specific anthocyanins (e.g., pelargonidin and delphinidin) are significantly positively correlated with ABTS radical scavenging activity, supporting the central role of phenolic compounds as electron donors in antioxidant reactions (Anantaworasakul et al. 2025). Furthermore, positive correlations were observed between processing‐derived compounds such as theaflavins and chlorogenic acid, as well as between theabrownins and rutin or theaflavins, illustrating the oxidative transformation pathway in which phenolics polymerize into more complex compounds during processing. Notably, certain organic acids—including gentisic acid and chlorogenic acid—showed negative or weak correlations with antioxidant metrics, challenging the generalized assumption that all phenolic acids enhance antioxidant activity. One plausible explanation is that, under Maillard reaction or thermal degradation conditions, these organic acids may not only lack direct antioxidant contribution but also consume more potent phenolic precursors, thereby indirectly reducing the overall antioxidant potential of the product (Wang, Peng, et al. 2025).

In contrast to antioxidant activity, α‐amylase inhibitory activity exhibited only weak correlations with most components measured in this study—a finding of considerable significance. This finding strongly suggests that the observed bioactivity is not primarily dictated by broadly quantified constituents like total polyphenols or flavonoids, but rather shows a stronger association with specific, unmeasured trace components—for instance, alkaloids with particular structures—or newly formed, high‐affinity compounds generated during processing, such as protein‐polyphenol complexes. These results highlight limitations in conventional quality assessment systems that rely heavily on total phenolic or flavonoid content as benchmarks. In summary, while total polyphenols and flavonoids constitute the foundational material basis for biological activity, our correlation analysis suggests that the dynamically changing specific secondary metabolites—such as particular aglycones and tea pigments—formed during processing may serve as key determinants of ultimate functional divergence. Consequently, we hypothesize that future research should extend beyond macro‐level compositional indicators to closely track the transformation dynamics of critical bioactive constituents throughout processing.

## 4 Conclusions

Using UPLC‐QToF‐MS‐based untargeted metabolomics, this study elucidated how processing reshapes the phytochemical profile and bioactivity of hawk tea. We putatively annotated 2610 metabolic features and clearly distinguished the metabolic signatures of green (GHT), yellow (YHT), and black hawk tea (BHT) through multivariate analysis. The degree of fermentation induced extensive metabolic reprogramming, which fundamentally altered the chemical architecture of the leaves. GHT showed the strongest antioxidant and α‐amylase inhibitory activities, attributable to its high retention of native phenolics. Although fermentation reduced some antioxidant components in BHT, it enriched specific metabolites that contribute to enzyme inhibitory effects. This research establishes a strong association between processing‐induced metabolic reprogramming and the functional differentiation of hawk tea, providing a metabolomic basis for its sensory and biofunctional divergence and supporting its targeted development as a high‐value functional beverage.

## Author Contributions

**Xiuming Zhai:** methodology. **Fuliang Xiao:** methodology. **Jie Li:** writing – original draft, investigation. **Yaxin Liu:** data curation. **Qiyang Chen:** conceptualization, writing – review and editing, funding acquisition. **Jinyan Zhao:** investigation, writing – original draft. **Dan Wang:** conceptualization, writing – review and editing, funding acquisition.

## Funding

This work was supported by Chongqing Modern Agricultural Industry Technology System Tea Innovation Team (CQMAITS202508) and Chongqing Technical Innovation and Application Development Special Project (cstc2021jscxgksbX0006).

## Conflicts of Interest

The authors declare no conflicts of interest.

## Supporting information


**Figure S1:** Score scatter plot of OPLS‐DA model for group GHT VS YHT (A), GHT VS BHT (B) and YHT VS BHT (C) and permutation test of OPLS‐DA model for group GHT VS YHT (D), GHT VS BHT (E) and YHT VS BHT (F). The model parameters for each comparison are as follows: GHT vs. YHT: *R* ^2^ *X* = 0.748, *R* ^2^ *Y* = 1, *Q* ^2^ = 0.948; GHT vs. BHT: *R* ^2^ *X* = 0.828, *R* ^2^ *Y* = 1, *Q* ^2^ = 0.988; YHT vs. BHT: *R* ^2^ *X* = 0.868, *R* ^2^ *Y* = 1, *Q* ^2^ = 0.983.

**Table S1:** All differentially expressed metabolites (292 species).
**Table S2:** Differential metabolites in the network diagram.
**Table S3:** Differentially metabolized compounds in the pathway diagram.

## Data Availability

The data that support the findings of this study are available on request from the corresponding author. The data are not publicly available due to privacy or ethical restrictions.
