# Supplementary material for: Processing‐Induced Metabolic Reprogramming of Litsea coreana Modulates Its Phytochemical Profile and Bioactivities
Source: Food Sci Nutr. 2026 May 13;14(5):e71880. doi: 10.1002/fsn3.71880 (PMC13172275; doi:10.1002/fsn3.71880)
Supplement: Supplementary file 1 — Figure S1: Score scatter plot of OPLS‐DA model for group GHT VS YHT (A), GHT VS BHT (B) and YHT VS BHT (C) and permutation test of OPLS‐DA model for group GHT VS YHT (D), GHT VS BHT (E) and YHT VS BHT (F). The model parameters for each comparison are as follows: GHT vs. YHT: R 2 X = 0.748, R 2 Y = 1, Q 2 = 0.948; GHT vs. BHT: R 2 X = 0.828, R 2 Y = 1, Q 2 = 0.988; YHT vs. BHT: R 2 X = 0.868, R 2 Y = 1, Q 2 = 0.983. [file FSN3-14-e71880-s001.docx]

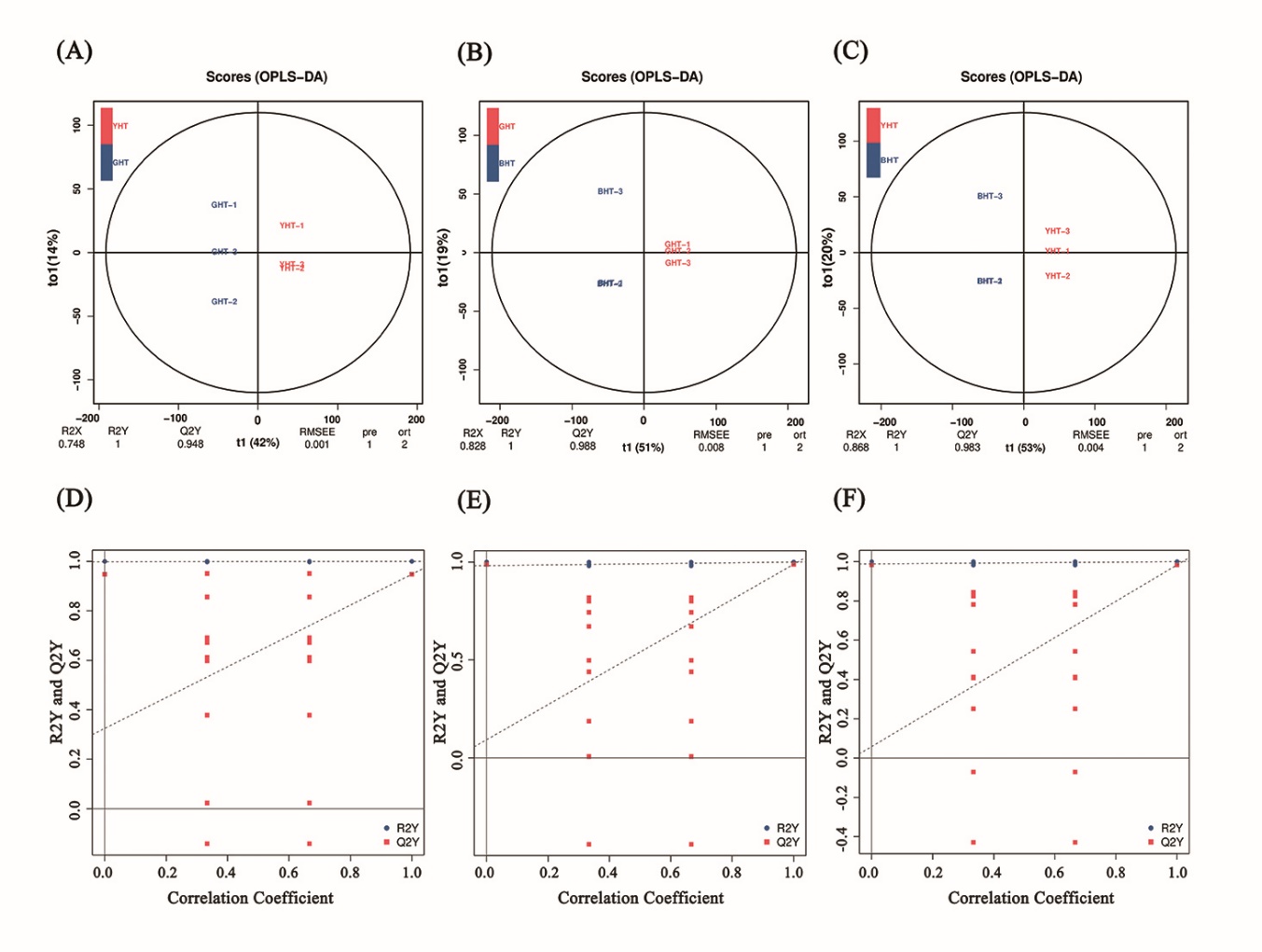


**Fig. S1** Score scatter plot of OPLS-DA model for group GHT VS YHT(A), GHT VS BHT(B) and YHT VS BHT (C)and permutation test of OPLS-DA model for group GHT VS YHT(D), GHT VS BHT(E) and YHT VS BHT(F). The model parameters for each comparison are as follows: GHT vs YHT: R²X = 0.748, R²Y = 1, Q² = 0.948; GHT vs BHT: R²X = 0.828, R²Y = 1, Q² = 0.988; YHT vs BHT: R²X = 0.868, R²Y = 1, Q² = 0.983.
